# Reduction in pediatric growth hormone deficiency and increase in central precocious puberty diagnoses during COVID 19 pandemics

**DOI:** 10.1186/s13052-022-01238-1

**Published:** 2022-03-28

**Authors:** Martina Peinkhofer, Benedetta Bossini, Arturo Penco, Manuela Giangreco, Maria Chiara Pellegrin, Viviana Vidonis, Giada Vittori, Nicoletta Grassi, Elena Faleschini, Egidio Barbi, Gianluca Tornese

**Affiliations:** 1 University of Trieste, Trieste, Italy; 2 Institute for Maternal and Child Health - IRCCS “Burlo Garofolo”, Via dell’Istria, 65/1, 34137 Trieste, Italy

**Keywords:** Stimulation test, Central precocious puberty, Growth hormone deficiency, COVID-19

## Abstract

**Background:**

While several studies have been published so far on the effect of COVID-19 pandemic on health care for non-COVID-19 diseases, to date no study evaluated the impact of the COVID-19 pandemic on the entire field of pediatric endocrinology. This study aimed to evaluate differences in pediatric endocrine stimulation tests after the advent of COVID-19 pandemics.

**Methods:**

Retrospective study with data collection for pediatric endocrine stimulation tests performed in 2019 and 2020 in a tertiary center.

**Results:**

Overall, 251 tests were performed on 190 patients in 2020, compared to 278 tests on 206 patients in 2019 (− 10% tests; − 8% children evaluated). A significant reduction was found in tests to diagnose growth hormone deficiency (GHD) (− 35%), while LHRH tests increased (+ 22%). A reduction of 30% in GHD diagnosis was observed. Central precocious puberty (CPP) diagnosis increased by 38% compared to 2019, mainly in females.

**Conclusion:**

This study found a significant reduction of tests investigating GHD during COVID-19 pandemics. It also showed a clinically meaningful increase in cases of CPP in girls. These results suggest the need for families and pediatricians to monitor children’s growth during isolation and enlighten new perspectives towards conditions associated with lockdown restrictions as increased screen time, social isolation, and children’s anxiety as possible triggers of CPP.

## Background

The two most frequent referral reasons in pediatric endocrinology are issues regarding growth and puberty, which constitutes almost half of the referrals for pediatric endocrinologists [1]. To detect growth hormone deficiency (GHD) or central precocious puberty (CPP), among other endocrine diseases, the determination of basal hormones is of limited diagnostic value, and stimulation tests – which require hospital admission – are needed. Although some cases of CPP could even be diagnosed by determining basal values of LH, FSH, and sexual hormones, stimulation tests are still extensively used and considered the gold standard in the diagnosis and help in diagnosing cases where basal values are not conclusive [2].

Due to the COVID-19 pandemic, from March 9th, Italy was placed under the first national generalized lockdown: people across the entire peninsula, with unprecedented measures, were ordered to stay at home, and travels were banned, excluding only those for “urgent, verifiable work situations and emergencies or health reasons.” To reduce pressure on hospitals and minimize the hazard of direct person-to-person exposure, non-urgent visits were reduced. Also, schools of all levels, from kindergarten to universities, were kept closed for physical attendance, while teaching was pursued by telematic means. All activities gradually resumed from May 4th to June 15th, 2020, and until October 26th, when Italian authorities introduced new restrictions nationwide until May 2021.

While several studies have been published so far on the effect of COVID-19 pandemic on health care for non-COVID-19 diseases, mainly in pediatric emergency departments, indicating a reduction in both urgent and non-urgent visits [3–11], to date, no study evaluated the impact of the COVID-19 pandemic on the entire field of pediatric endocrinology.

This study aimed to evaluate differences between 2019 and 2020 in pediatric endocrine stimulation tests performed in a tertiary hospital.

## Material and methods

We conducted a retrospective study at the Institute for Maternal and Child Health IRCCS “Burlo Garofolo” in Trieste, Italy, a tertiary hospital and research institute that serves as a pediatric referral center for the province of Trieste and as a national reference hospital.

During COVID-19 pandemics, admissions for endocrine stimulation tests were among the procedures that were considered urgent and therefore not canceled. The available number of beds for investigations remained unchanged to 300 per year. All records of children and adolescents performing a stimulation test from January 1st to December 31st, 2019 [2] and from January 1st to December 31st, 2020, were reviewed. The “G2 clinico” platform (management system specialist activities) was employed to access all patients’ data. Information retrieved included age at presentation, gender, height SDS, BMI SDS, type and number of tests performed, reason(s) for referral, test results, and final diagnosis. Stimulation tests were performed and interpreted as previously described [2].

Ethical Committee approval was not requested since General Authorization to Process Personal Data for Scientific Research Purposes (Authorization no. 9/2014) declared that retrospective archive studies that use ID codes, preventing the data from being traced back directly to the data subject, do not need ethics approval [12]. Parents signed informed consent at the first visit, in which they agreed that “clinical data may be used for clinical research purposes, epidemiology, the study of pathologies and training, to improve knowledge, care, and prevention.”

Statistical analyses were mainly descriptive. Data are presented as frequencies and percentages or as median and interquartile ranges (IQRs) due to non-normal distribution. Fisher’s exact test was used to evaluate associations between two categorical variables, while the non-parametric Wilcoxon Mann-Whitney test was applied to assess the difference in the distribution of a continuous variable across the two groups of a categorical variable. A *P*-value < 0.05 was considered statistically significant. Analyses were performed with JMP™ software (version 16.1.0, SAS Institute Inc., Cary, NC, United States).

## Results

Overall, while in 2019 278 tests were performed on 251 patients in 2019 (47% females) with a median age of 11.1 years (IQR 8.0;14.0), in 2020 251 tests were performed on 190 patients (66% females, *p* < 0.05) with a median age of 12.1 years (IQR 8.1;15.1); 59 individuals (29%) in 2019 and 48 individuals (25%) in 2020 performed more than 1 test.

The distribution of stimulation tests and pathological response is reported in Table 1*.* A significant difference among performed tests was found between 2019 and 2020 (*p* < 0.01): while in 2019 the most frequently performed test was Arginine Tolerance Test (ATT: *n* = 95, 34%), in 2020 it was Luteinizing Hormone Releasing Hormone (LHRH) Test (LHRHT: *n* = 81, 33%).

**Table 1:** Distribution of endocrine stimulation test according to suspected diagnosis and pathological tests in 2019 and 2020 (^a^ ITT were performed to detect both GHD and CAI simultaneously)

		2019			2020		
**Suspected diagnosis**	**Stimulation test**	**N of performed tests**	**N of pathological tests**	**% of pathological tests**	**N of performed tests**	**N of pathological tests**	**% of pathological tests**
GHD	ATT	95	39	41%	60	25	42%
	ATT + GHRHT	1	1	100%			
	CTT				1	1	100%
	ITT^a^	31	25	81%	19	16	84%
NC-CAH	SDST	52	0	0%	56	1	2%
CPP	LHRHT	48	24	50%	54	28	52%
CAI	ITT^a^	31	5	16%	19	4	21%
	LDST	16	1	6%	13	1	8%
	CRHT				1	0	0%
HH	LHRHT	18	5	28%	27	1	4%
AGHD	ATT + GHRHT	17	0	0%	15	1	7%
CH	TRHT				1	1	100%
OG	OGTT				4	2	50%

*AGHD* Adult Growth Hormone Deficiency, *ATT* Arginine Tolerance Test, *ATT + GHRHT* Arginine Tolerance Test + Growth Hormone Releasing Hormone Test, *CAI* Central Adrenal Insufficiency, *CH* Central Hypothyroidism, *CPP* Central Precocious Puberty, *CRHT* Corticotropin-Releasing Hormone Test, *CTT* Clonidine Tolerance Test, *GHD* Growth Hormone Deficiency, *HH* Hypogonadotropic Hypogonadism, *ITT* Insulin Tolerance Test, *LDST* Low-Dose Synacthen Test, *LHRHT* Luteinizing Hormone-Releasing Hormone Test, *NC-CAH* Non-Classical Congenital Adrenal Hyperplasia, *OG* OverGrowth, *OGTT* Oral Glucose Tolerance Test, *SDST* Standard-Dose Synacthen Test, *TRHT* Thyrotropin Releasing Hormone

### GHD tests

**Fig. 1:**
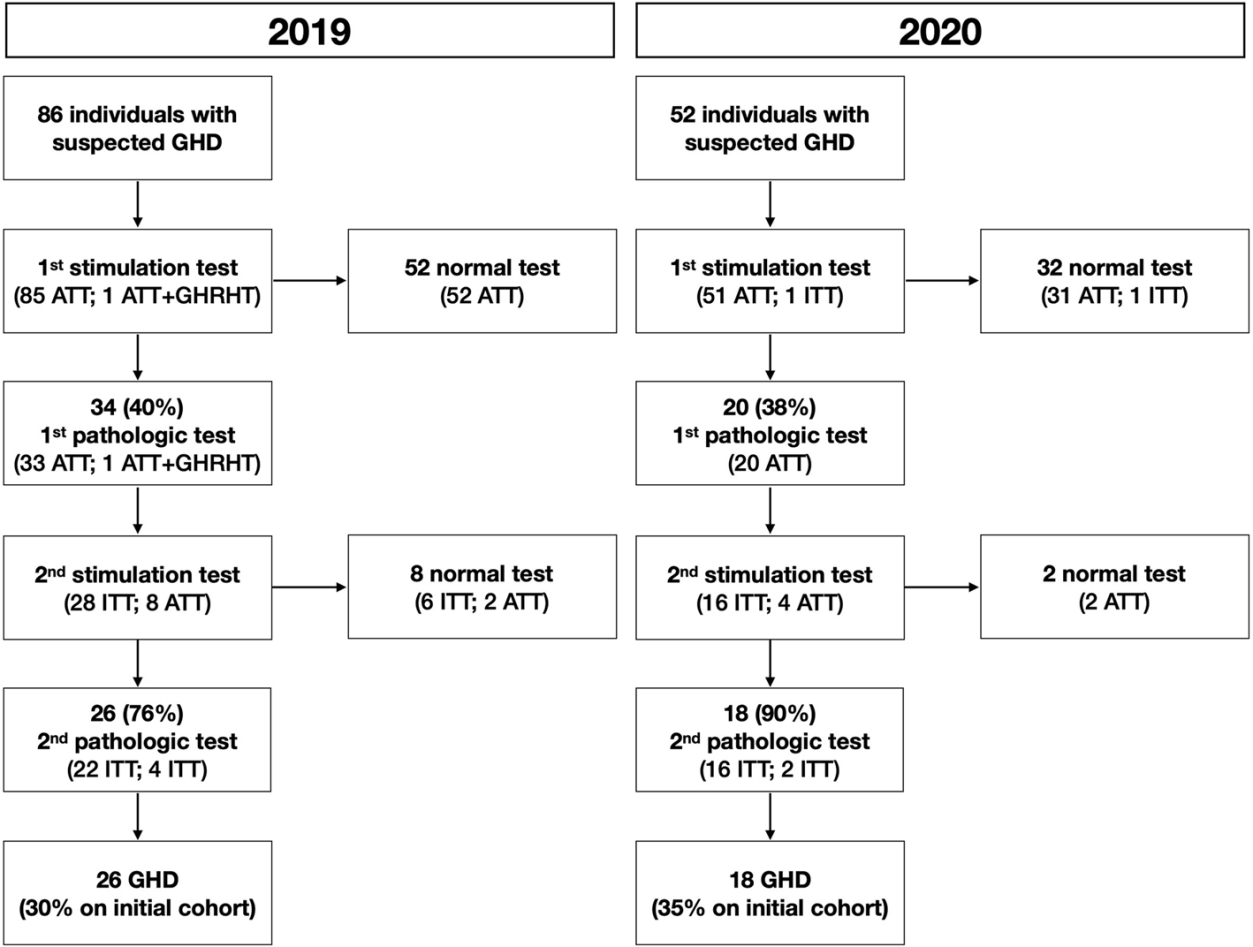
Diagnostic process for children with suspected growth hormone deficiency (GHD) in 2019 and 2020

Among tests evaluating a suspected GHD (arginine [ATT], insulin [ITT], and clonidine [CTT] tolerance tests), a total reduction from 126 to 80 was detected (Table 1): 86 children were investigated for short stature in 2019 and 52 in 2020 (*p* < 0.01), and GHD was diagnosed in 26 children in 2019 and 18 in 2020 (*p* = 0.02) (Fig. 1)*.* Median age at first stimulation test was 12.0 years (IQR 8.7;13.0) in 2019 and 12.5 years (IQR 9.0;13.5) in 2020 (*p* = 0.24). Overall, in 2020 there was a 40% reduction of patients evaluated for a suspected GHD with a decrease of 30% in diagnoses of GHD compared to 2019, while the rate of positive tests was similar in the 2 years (30% in 2019, 35% in 2020) (*p* = 0.81). No differences were found in height SDS at first stimulation test between 2019 (median − 2.20 [IQR -2.57;-1.79]) and 2020 (median − 2.17 [IQR -2.71;-1.46]) (*p* = 0.91).

### LHRH tests

Details regarding LHRH tests are provided in Table 2. The median age at test was 7.8 years (IQR 7.1;8.5) for females and 9.0 years for males (IQR 8.8;9.6). An increase of tests for a suspect of CPP in 2020 (+ 13%) was associated with a rise in pubertal responses (+ 17%) compared to 2019. Considering tests performed for suspected CPP, the number of diagnoses in females was higher in 2020 than in 2019: 16 diagnoses in 2019 (42% of tests) vs. 26 diagnoses in 2020 (58% of tests) (*p* = 0.03). No differences were found in age between the two years for females (*p* = 0.26) and males (*p* = 0.74).

**Table 2:** Details on LHRH tests performed in 2019 and 2020

		***2019***			***2020***		
		Normal	Pathological	Total	Normal	Pathological	Total
Suspected diagnosis	**CPP**	24 (50%)	24 (50%)	48	26 (49%)	28 (51%)	54
	*Females*	*22*	*16*	*38*	*19*	*26*	*45*
	*Males*	*2*	*8*	*10*	*7*	*2*	*9*
	**HH**	13 (73%)	5 (27%)	18	26 (96%)	1 (4%)	27
	**Total**	**66**			**81**		

*CPP* Central Precocious Puberty, *HH* Hypogonadotropic Hypogonadism

There was not a significant difference in BMI SDS for children with a suspect of CPP between 2019 (median 0.45 [IQR -0.16;1.01]) and 2020 (median 0.45 [IQR -0.64;0.89]). While no significant differences were found in BMI SDS between all children diagnosed with CPP in 2019 (median 0.46 [IQR 0.13;1.01]) and in 2020 (median 0.17 [− 0.30;0.74]) (*p* = 0.15), females diagnosed with CPP in 2020 had a BMI SDS (median 0.11 [IQR -0.52;0.72]) significantly lower than those diagnosed in 2019 (median 0.93 [IQR 0.38;1.10]) (*p* = 0.01).

## Discussion

In this retrospective study, we comprehensively analyzed differences in endocrine stimulation tests performed in a pediatric endocrinology outpatient department between 2019 and 2020 to detect any effect of COVID-19 pandemic restrictions.

Overall, we found a reduction of 10% in tests and 8% in children evaluated between 2019 and 2020, which was not related to hospital policies, since endocrine stimulation tests were considered urgent admissions during COVID-19 lockdown and the available number of beds for investigations remained unchanged. The most significant detected changes were a reduction in tests to diagnose GHD (− 30% of patients diagnosed in 2020) and an increase in CPP diagnosis (+ 38%, mainly females).

Regarding the tests investigating GH secretion, we detected a striking reduction (− 35%) in tests performed in 2020, compared to 2019. This was sharply in contrast with a steady increase in referrals for growth issues that we reported from 2014 to 2018 in a previous study [1], even though the criteria – according to the Italian Medicines Agency (AIFA) – to perform stimulation tests were the same (i.e., height ≤ − 3 SD; or height ≤ − 2 SD + growth velocity per year ≤ − 1 DS for age and sex evaluated for at least 6 months or a reduction of height of 0.5 SD per year in children > 2 years of age; or height ≤ − 1.5 SD compared to target height and growth velocity per year ≤ − 2 SD or ≤ − 1.5 SD for two consecutive years; or growth velocity per year ≤ − 2 SD or ≤ − 1.5 SD after two consecutive years, even without short stature and after exclusion of other causes of failure to thrive; or malformations/lesions of hypothalamus/pituitary demonstrated at brain MRI) [13]. Although this is the first study reporting a decrease of referrals for suspected GHD during 2020 lockdowns, our results align with previous reports addressing a delay of hospital care admissions and a reduction of 30–40% in visits during the COVID-19 pandemic [9, 10]. A possible explanation for the decrease in tests for suspected GHD is that well-child visits were canceled during the pandemics, removing a chance for family pediatricians to detect short stature and delayed growth. Moreover, families did not have the opportunity to compare their children with classmates (because of remote schooling and the absence of other social activities), missing possible deviations from the regular growth pattern to share with their family pediatricians. Although not considered in the present study, we have to contemplate that some families might have canceled their booked appointment because they feared going to the hospital during a pandemic or thought short stature was not an urgent issue. Since the rate of GHD among referred children was similar in the 2 years (30% in 2019, 35% in 2020) and coherent with previous data [1, 14] we probably missed 8 GHD diagnoses in 2020. Although these children might have been referred and eventually treated at a later stage, we know that the efficacy of treatment is increased if started at a younger age [15]. Moreover, approximately 35% of the patients with GHD have an organic etiology, and a delay in such a diagnosis can be life-threatening [16].

On the other side, our study found a clinically meaningful increase in CPP diagnoses in girls (+ 16%) during 2020 compared to the same period in 2019. A “breast bud” appearance in a girl before the age of 8 years is always troublesome and easier to detect by families, even without comparisons with peers. Moreover, parents working from home might have increased the time spent with their children, detecting early pubertal changes more quickly. On the other hand, there was a decrease of 75% in CPP diagnosis in males. This reduction can be determined by the subtle finding of testicular enlargement (the earliest evidence of puberty in males) that patients and parents often go unnoticed and can be missed if well-child visits from family pediatricians are missed.

An increase in the incidence of CPP had already been reported years before the advent of the COVID-19 pandemic in several countries, including France (Gaspari L, et al. [17]), Korea (Kim SH et al., [18])and Denmark (Sømod ME, et al. [19]), but not Italy where both referrals for CPP [1] and new diagnosis of CPP (Stagi [20]) were stable during the previous 5 years.

Our findings of an increase of CPP during COVID-19 lockdown are in accordance with other two Italian reports: the first study found an increased incidence of newly diagnosed CPP in females with a faster rate of pubertal progression during COVID-19 pandemics compared to the same period, each year, from 2015 to 2019 [20]; similarly, in the second study, a rise in referrals for suspected precocious puberty was recorded in 2020 (+ 108% consultations compared to 2019) [21]. Since the hypothesis of a direct effect of Sars-CoV2 as a trigger of puberty remains unlikely, these results seem to suggest a possible role of environmental factors on the early onset of puberty during COVID-19 pandemics [22].

Precocious pubertal timing is crucial, resulting in a child sexually mature at an emotionally and socially inappropriate age, thus leading to risk-taking behaviors as sexual relations and substance use [23]. Genetics plays a significant role in CPP; however, environmental factors as obesity and adverse childhood experiences may influence pubertal development [24, 25]. Moreover, epidemiological studies in humans demonstrated that exposures to endocrine-disrupting compounds have pronounced effects on pubertal timing increasing an early onset of puberty [26]. Since COVID-19 outbreak limitations forced children to home-schooling and sedentary lifestyle, a cross-sectional study found a change in dietary habits, with increased consumption of hypercaloric foods as pizza, bread, sweets, and more snacking, thus leading to overweight [27]. Considering these observations, we hypothesized a possible association of increased overweight secondary to COVID-19 restrictions and early puberty. Interestingly, we found no increase in BMI SDS between early pubertal girls observed in 2020. Our results align with an Italian report on referrals for precocious puberty in 2020, where no significant differences in anthropometric parameters were found compared to 2019 [21]. COVID-19 restrictions exposed children to e-learning and increased the use of screens. Stagi et al. found a more than doubled increase in time spent using electronic devices in children during the lockdown, hypothesizing its possible role in triggering puberty’s early onset and tempo [20].

A potential limitation of this study is based on data collected retrospectively from a single center; therefore, results may be related to the local population and did not allow further analysis on other contributing factors. On the other hand, to our knowledge, this is the first study that has simultaneously analyzed the features of pediatric endocrine stimulation tests as a whole during COVID-19 pandemics. Another limitation is the comparison between 2 years (2019 and 2020) only, while it could have been interesting to analyze data from the previous years, as well as it could be interesting to evaluate data in the following years to evaluate if the founded changes were part of a trend from the previous years or were limited to the lockdown restrictions or due to the change in everyday life during pandemic years.

## Conclusion

This study found a significant reduction of tests investigating GHD during COVID-19 pandemics, suggesting that a considerable number of children with GHD may have missed the opportunity of a timely diagnosis. It also showed a clinically meaningful increase in cases of CPP in girls. These results suggest the need for families and pediatricians to monitor children’s growth during isolation and enlighten new perspectives towards conditions associated with lockdown restrictions as increased screen time, social isolation, and children’s anxiety as possible triggers of CPP.

Since COVID-19 continues to represent a major global health threat challenging the accessibility to healthcare services, clinicians and the public health system should be aware that reduced access to health care is detrimental to both diagnosis and treatment of endocrine conditions.

## Data Availability

The data that support the findings of this study are available from the corresponding author, GT, upon reasonable request.

## Abbreviations

AGHD: Adult Growth Hormone Deficiency
AIFA: Italian Medicines Agency
ATT: Arginine Tolerance Test
ATT + GHRHT: Arginine Tolerance Test + Growth Hormone Releasing Hormone Test
BMI SDS: Body Mass Index Standard Deviation Score
CAI: Central Adrenal Insufficiency
CH: Central Hypothyroidism
CPP: Central Precocious Puberty
*CRHT*: *Corticotropin-Releasing Hormone Test*
*CTT*: *Clonidine Tolerance Test*
GHD: Growth Hormone Deficiency
HH: Hypogonadotropic Hypogonadism
IQR: InterQuartile Ranges
ITT: Insulin Tolerance Test
LDST: Low-Dose Synacthen Test
LHRHT: Luteinizing Hormone-Releasing Hormone Test
NC-CAH: Non-Classical Congenital Adrenal Hyperplasia
OG: OverGrowth
OGTT: Oral Glucose Tolerance Test
SDST: Standard-Dose Synacthen Test
TRHT: Thyrotropin Releasing Hormone
